# Influence of Gallic Acid-Containing Mouth Spray on Dental Health and Oral Microbiota of Healthy Cats—A Pilot Study

**DOI:** 10.3390/vetsci9070313

**Published:** 2022-06-22

**Authors:** Chaiyavat Chaiyasut, Bhagavathi Sundaram Sivamaruthi, Muruganantham Bharathi, Chawin Tansrisook, Sartjin Peerajan, Khontaros Chaiyasut, Suchanat Khongtan, Kittidaj Tanongpitchayes, Nichaphat Thongma, Natcha Chawnan, Kriangkrai Thongkorn

**Affiliations:** 1 Innovation Center for Holistic Health, Nutraceuticals, and Cosmeceuticals, Faculty of Pharmacy, Chiang Mai University, Chiang Mai 50200, Thailand; chaiyavat@gmail.com (C.C.); bharathi.m@cmu.ac.th (M.B.); chawin2007@gmail.com (C.T.); suchanat_k@cmu.ac.th (S.K.); kittidaj_tanong@cmu.ac.th (K.T.); 2 Office of Research Administration, Chiang Mai University, Chiang Mai 50200, Thailand; 3 Health Innovation Institute, Chiang Mai 50200, Thailand; s.peerajan@gmail.com; 4 Institute of Research and Development, Chiang Mai Rajabhat University, Chiang Mai 50300, Thailand; khontaros_cha@cmru.ac.th; 5 Small Animal Hospital, Faculty of Veterinary Medicine, Chiang Mai University, Chiang Mai 50200, Thailand; nichaphat_thongma@cmu.ac.th; 6 Department of Companion Animal and Wildlife Clinic, Faculty of Veterinary Medicine, Chiang Mai University, Chiang Mai 50100, Thailand; natcha_chaw@cmu.ac.th

**Keywords:** gallic acid, mouth spray, cats, oral microbiome, oral hygiene, gingival index, plaque index

## Abstract

**Simple Summary:**

Periodontal diseases are common dental issues in cats. Oral care supplements were used to prevent diseases and maintain oral health. Moreover, maintaining a healthy oral microbiome is crucial for oral health. Therefore, we have developed a gallic acid-containing mouth spray and studied its effect on oral microbiota and dental health in healthy cats. The results revealed that the gingival and plaque indexes were improved after 42 days of mouth spray treatment in cats. The mouth spray treatment also reduced the abundance of harmful bacterial load and supported the growth of normal oral microbiota. This preliminary study recommended that the gallic acid-containing mouth spray could be an essential oral product to improve the oral hygiene of the cats.

**Abstract:**

This pilot study aimed to investigate the effects of gallic acid-containing mouth spray on oral microbiota in healthy cat subjects. Forty healthy cats were recruited and randomly allocated to the control (G1; *n* = 20) and treatment groups (G2; *n* = 20). The cats were treated with mouth spray twice daily for 42 days. The changes in the gingival index (GI) and plaque index (PI) were measured at baseline (day 0) and end of the study (42nd day). The changes in the oral microbial composition of representative animals (control, *n* = 9; and treatment, *n* = 8) were also evaluated at baseline and end of the study. Oral microbial composition was assessed by amplifying the V1–V3 region of the 16S rRNA gene from supragingival dental plaque DNA extracts. The sequences were annotated using the QIIME 2.0. The GI and PI were significantly reduced after 42 days of treatment. The deep sequencing revealed that mouth spray influenced the cats’ oral microbiome and was significantly diverse. About 20 phyla and 59 species were observed after 42 days of mouth spray usage in cats’ oral microbiota. The number of operational taxonomic units (OTUs) of post-treatment samples (PoTS) of G2 was greatly reduced compared to other samples. Further analysis revealed that mouth spray acts substantially against *Desulfomicrobium orale*, one of the known pathogens in periodontal disease. The mouth spray efficiently reduced the growth of 22 species and uprooted 17 species. Moreover, the mouth spray supported the growth of normal oral microbiota, including *Moraxella* and *Neisseria* species. The preliminary study suggested that the gallic acids-containing mouth spray could be an essential oral product to improve the oral hygiene of the cats. Moreover, further studies are needed to confirm the beneficial effect of mouth spray on cats.

## 1. Introduction

The oral microbiome is involved in the health and diseases of cats [1]. Although several studies deal with the microbiome, very few studies concentrate on the cat’s oral microbiota. In cats, oral cavities are the most identified health concern [2,3]. About 50–90% of cats suffer from dental diseases [4]. Periodontal disease is the most prevalent dental disorder in cats, and the severity of each of these problems varies greatly. Periodontal disease starts with gingivitis, mild irritation, and inflammation in the gingiva, followed by periodontitis. It is an inflammation in periodontal tissues, accumulation of immune cells, chronic oral infection, bacteremia, and tooth loss [5,6]. Dental calculus thickness, calculus coverage, and anaerobic bacterial infection are associated with the severity of gingivitis [7]. The factors associated with feline chronic gingivostomatitis are debatable, and feline-specific calicivirus, herpesvirus, immunodeficiency virus, and leukemia virus are involved in the development of the disease [8].

The studies suggested that the use of oral care supplements could help prevent dental diseases in cats. The addition of dental chews in the dry diet effectively reduced the plaque and calculus accumulation and reduced the severity of gingivitis in cats [9]. Clarke evidenced that zinc ascorbate gel may be most efficient in lowering bacterial development, plaque formation, and gingivitis. It can be administered as an oral disinfectant to enhance oral health combined with professional teeth cleaning [10]. According to Rawlings et al., chlorhexidine would have been the most efficient compound in reducing gingivitis and accumulating dental plaque in canines [11].

Gallic acid is widely present in various plants, fruits, and nuts, and it has been recognized for its several biological activities, including antioxidant, antimicrobial, and anti-inflammatory activity [12]. Gallic acid could regulate the intracellular mitogen-activated protein kinase and nuclear factor kappa-light-chain-enhancer of activated B cells (NF-κb) pathways, and it reduced the expression of tumor necrosis factor-alpha and interleukin-6 [12].

Concerning oral health, gallic acid suppressed the growth of cariogenic pathogens and especially inhibits *Streptococcus mutans* biofilm formation [13]. Recently, Karatas and Gevre reported that gallic acid treatment (30 and 60 mg per kg) significantly reduced the alveolar bone loss, inflammation, and tartrate-resistant acid phosphatase (TRAP) positive osteoclast cell count in the Wister rat periodontitis model. Moreover, the expression of matrix metalloproteinase (MMP)-8 was decreased, and the expression of bone morphogenetic protein-2 and tissue inhibitor of MMPs was increased in the gallic acid treatment group compared to the disease control group [14]. Gallic acid reduces microbial colonization on abiotic surfaces by inhibiting bacterial adhesion and biofilm development, possibly by affecting the physicochemical properties of the cell surface and calcium efflux [15].

Developing our insights into the oral cat microbiota will undoubtedly help attempts to enhance cat oral health [16]. Understanding the species comprising the oral microbiome of the cat is important because the feline oral microbiome has a profound influence on cats’ oral and systemic health. Dewhirst et al. developed and presented the 16S rRNA gene reference set for the feline oral microbiome [17]. Subsequently, Nakanishi et al. reported that feline calicivirus load was higher in feline gingivostomatitis and the oral microbiome of the diseased cat was disrupted [18]. The feline chronic gingivostomatitis samples were reported for the enrichment of *Malassezia restricta*, *M. arunalokei*, *Cladosporium penidielloides*, and *Aspergillaceae* sp. Further, *Bergeyella zoohelcum* could be found as a biomarker for feline healthy oral microbiome [19]. *Porphyromonas*, *Moraxella*, *Fusobacterium* genera, Xanthomonadaceae family, and *Capnocytophaga canimorsus* and *Bergeyella* species are the most predominant microbial community of healthy cats [20,21]. The phyla Spirochaetes and Bacteroidetes are the key pathogens in cats’ oral diseases, such as gingivitis and mild periodontitis [14]. Another research found that *Pasteurella multocida* subsp. *multocida* was considerably common in infected cats [22].

Therefore, the present study aimed to investigate the gingival parameters, such as gingival index (GI) and plaque index (PI), and changes in the oral microbial community of healthy cats treated with gallic acid-containing mouth spray.

## 2. Materials and Methods

### 2.1. Preparation of Mouth Spray

The mouth spray was prepared with gallic acid (0.8%) (Merck, Darmstadt, Germany), ZnCl_2_ (0.1%) (Carlo Erba, Val-de-Reuil, France), hesperidin methyl chalcone (0.1%) (Merck, Darmstadt, Germany), poloxamer188 (10%) (BASF, Ludwigshafen, Germany), poloxamer407 (7.5%) (BASF, Ludwigshafen, Germany), carrageenan (0.2%) (Chemipan Corporation Co., Ltd., Bangkok, Thailand), sodium metabisulfite (0.5%) (Merck, Darmstadt, Germany), and water (80.9%). Carrageenan, poloxamer188, and poloxamer407 were added as mucoadhesive and thermosensitive gel-forming agents. Sodium metabisulfite served as an antioxidant.

### 2.2. Study Population and Design

The experimental cats were handled following the Good Animal Practice. The study was approved by the Animal Care and Use Committee (Ref. No. R2/2564), Faculty of Veterinary Medicine, Chiang Mai University, Chiang Mai, Thailand. Six weeks of experimental design were employed using healthy cats.

The inclusion and exclusion criteria of cat subjects are as follows [23]. The cats (5–8 years old) without any serious oral diseases and not on any kind of medications were included in the study. The cats with elevated blood urea nitrogen (BUN) and creatinine, alanine aminotransferase (ALT), and aspartate aminotransferase (AST) levels with more than two times upper normal limits were excluded from the study. Moreover, cats on medications were excluded from the study. The basic information about the experimental cats is described (Table 1).

Forty cats were selected and ambiguously assigned as control (G1; *n* = 20) and treatment groups (G2; *n* = 20). The cats were treated with mouth spray regularly twice a day for 42 days. All clinical parameters were assessed at baseline (pretreatment) and end of the study (day 42; post-treatment). The saliva was collected on day 0 and day 42 of the study from the representative subjects (control, *n* = 9; and treatment, *n* = 8) to evaluate the variations in oral microbiota.

### 2.3. Gingival Index (GI) and Plaque Index (PI)

For the study, eight teeth were selected from each cat (code no. 104, 108, 204, 208, 304, 309, 404, and 409). The TRIDAN modified method was utilized for tooth nomenclature [24].

GI and PI values of each tooth were measured at the baseline and end of the study. The results were represented as median and interquartile values of 20 cats.

GI was measured by inserting the periodontal probe into the periodontal pocket. The level of severity of GI was recorded (level 0 = normal gingival; level 1 = mild inflammation, no swelling, and no bleeding while inserting the periodontal probe into a periodontal pocket; level 2 = moderate inflammation, gingival redness, swelling, and bleeding while inserting the periodontal probe into a periodontal pocket; level 3 = severe inflammations and bleeding without insertion of a periodontal probe [7]).

PI was determined using the dental plaque disclosing gel (GC Tri plaque ID Gel^™^, GC America Inc., Alsip, IL, USA) per the manufacturer’s instructions. The stained area (%) was categorized by visual inspection (level 0 = no staining; level 1 = staining 33%; level 2: staining 66%; level 3: staining 100%) [25].

### 2.4. Next-Generation Sequencing (NGS)

The QIAamp UCP DNA Micro Kit was used to isolate the genomic DNA from saliva as per the manufacturer’s instructions (QIAGEN, Hilden, Germany). As detailed in our previous study, the Omics Sciences and Bioinformatics Center, Faculty of Science, Chulalongkorn University, performed the metagenomic analysis [26].

### 2.5. Statistical Analysis and Visualization

Fisher’s exact test was used to evaluate the differences in gender of the subjects. Mann–Whitney U test was used to evaluate the differences in weight and age of the experimental cats. Wilcoxon signed-rank test was used to assess the variations among the studied parameters (GI and PI). The changes were taken as significant if the *p* < 0.05. The values were represented as median and interquartile ranges.

The weighted and unweighted UniFrac distances were determined in the QIIME2 to compare the microbial richness of G1 and G2. The coordinates (initial major three) were utilized to produce PCoA plots, and they were labeled corresponding to their variance. The raw OUT counts were assimilated. The relative abundances and taxa of identical operational taxonomic units (OTUs) were taxonomically categorized. The taxonomical analysis of pretreatment samples (PrTS) and post-treatment samples (PoTS) of G1 and G2 and a separate comparison between PoTS were performed. The rarefaction curve was used to interpret the phylogenetic diversity (PD). The Shannon diversity index was also calculated, and a separate comparison between PoTS of G1 and G2 groups was performed to estimate the diversity of species. PCoA was used to correlate the samples and viewed in QIIME2.

## 3. Results

### 3.1. Changes in Gingival Index (GI) and Plaque Index (PI)

Cats’ gender, weight, and age were represented in Table 1, and there was no significant difference in it.

The GI and PI were measured for eight teeth in both groups (G1 and G2) on day 0 (pre) and day 42 (post). The GI and PI were analyzed. The pre-and post-treatment values were compared. All the parameters (GI and PI) were measured as ordinal data and represented as median and interquartile (Table 2). GI was significantly reduced in one tooth (108) in the control, whereas tooth no. 108 (*p* = 0.0003), 208 (*p* = 0.0111), 304 (*p* = 0.0327), 309 (*p* = 0.0122), and 409 (*p* = 0.0196) showed significant reduction compared to baseline in the treatment group (G2) (Table 2). Similarly, PI values were not improved in any of the teeth in the control (G1). Tooth no. 104 (*p* = 0.0497), 108 (*p* = 0.0154), 204 (*p* = 0.0084), 304 (*p* = 0.0257), 309 (*p* = 0.0298), and 409 (*p* = 0.0375) showed significant reduction compared to baseline in the treatment group (G2) (Table 2).

### 3.2. Oral Microbiome Analysis

#### 3.2.1. Taxonomy Assignment

**Figure 1:**
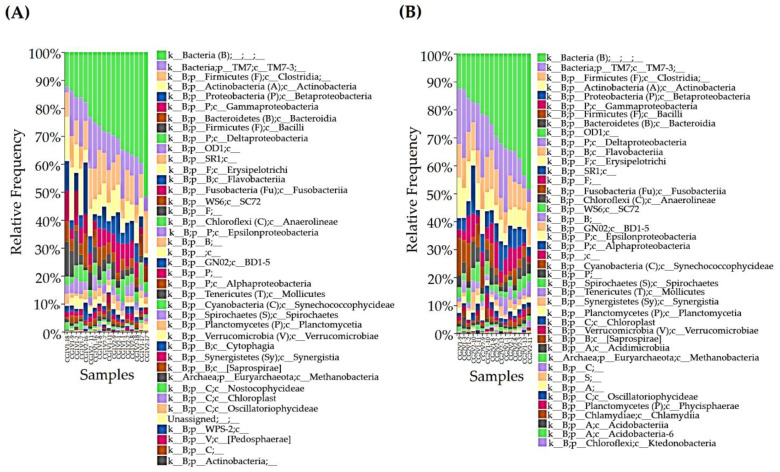
Taxonomical distribution of oral microbiome of experimental cats. (**A**) The comparison of PrTS and PoTS of G1 samples. (**B**) The comparison of PrTS and PoTS of G2. The relative frequency of the sample was compared. PrTS: pretreatment samples; PoTS: post-treatment samples.

A total of 67,848 and 81,420 microbial sequences were obtained from the PrTS and PoTS of G1, respectively. Likewise, 61,335 and 72,914 microbial sequences were obtained from PrTS and PoTS of G2. The sequences were grouped into OTUs using QIIME 2^TM^ (Figure 1).

#### 3.2.2. Phylum

**Figure 2:**
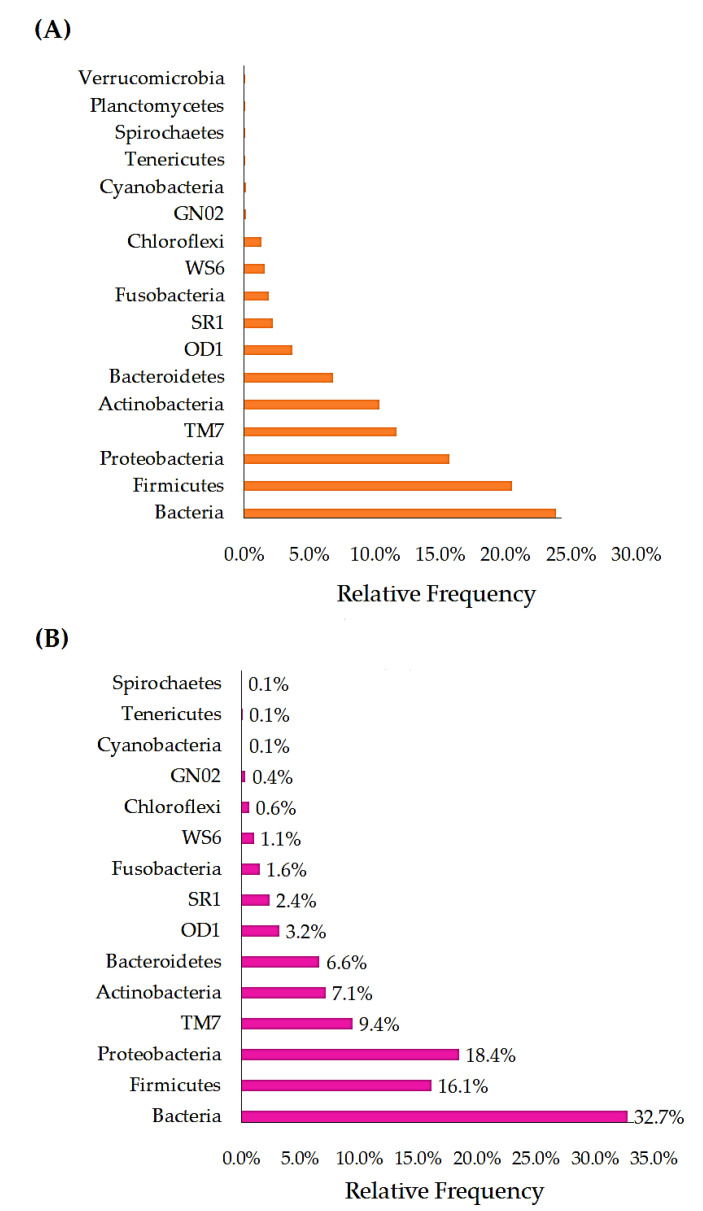
Comparison of the phylum-level relative frequency of PrTS (**A**) and PoTS (**B**) of the control group (G1). PrTS: pretreatment samples; PoTS: post-treatment samples.

The phyla Proteobacteria (μ = 15.7%), TM7 (μ = 11.7%), Actinobacteria (μ = 10.3%), Bacteroidetes (μ = 6.8%), OD1 (μ = 3.7%), SR1 (2.2%), Fusobacteria (μ = 1.9%), WS6 (μ = 1.6%), Chloroflexi (μ = 1.3%), GN02 (μ = 0.2%), Cyanobacteria (μ = 0.1%), Spirochaetes (μ = 0.1%), Planctomycetes (μ = 0.1%), and Verrucomicrobia (μ = 0.1%) were identified in the PrTS of G1 (Figure 2A). Likewise, the phyla Firmicutes (μ = 16.1%), Proteobacteria (μ = 18.4%), TM7 (μ = 9.4%), Actinobacteria (μ = 7.1%), Bacteroidetes (μ = 6.6%), OD1 (μ = 3.2%), SR1 (μ = 2.4%), Fusobacteria (μ = 1.6%), WS6 (μ = 1.1%), Chloroflexi (μ = 0.6%), GN02 (μ = 0.4%), Cyanobacteria (μ = 0.1%), and Spirochaetes (μ = 0.1%) were observed in the PoTS of G1. However, Planctomycete and Verrucomicrobia were not detected in the PoTS of G1. The relative frequency of unclassified bacterial lineage (μ = 32.7%) was increased in the PoTS compared to PrTS of G1 (μ = 23.83%). Firmicutes (μ = 20.5%) was found majorly in the PrTS (Figure 2A), whereas the amount of Firmicutes (μ = 16.14%) was decreased in the PoTS (Figure 2B) of G1. Moreover, the phylum Tenericutes (μ = 0.1%) was newly evolved in the PoTS of G1.

**Figure 3:**
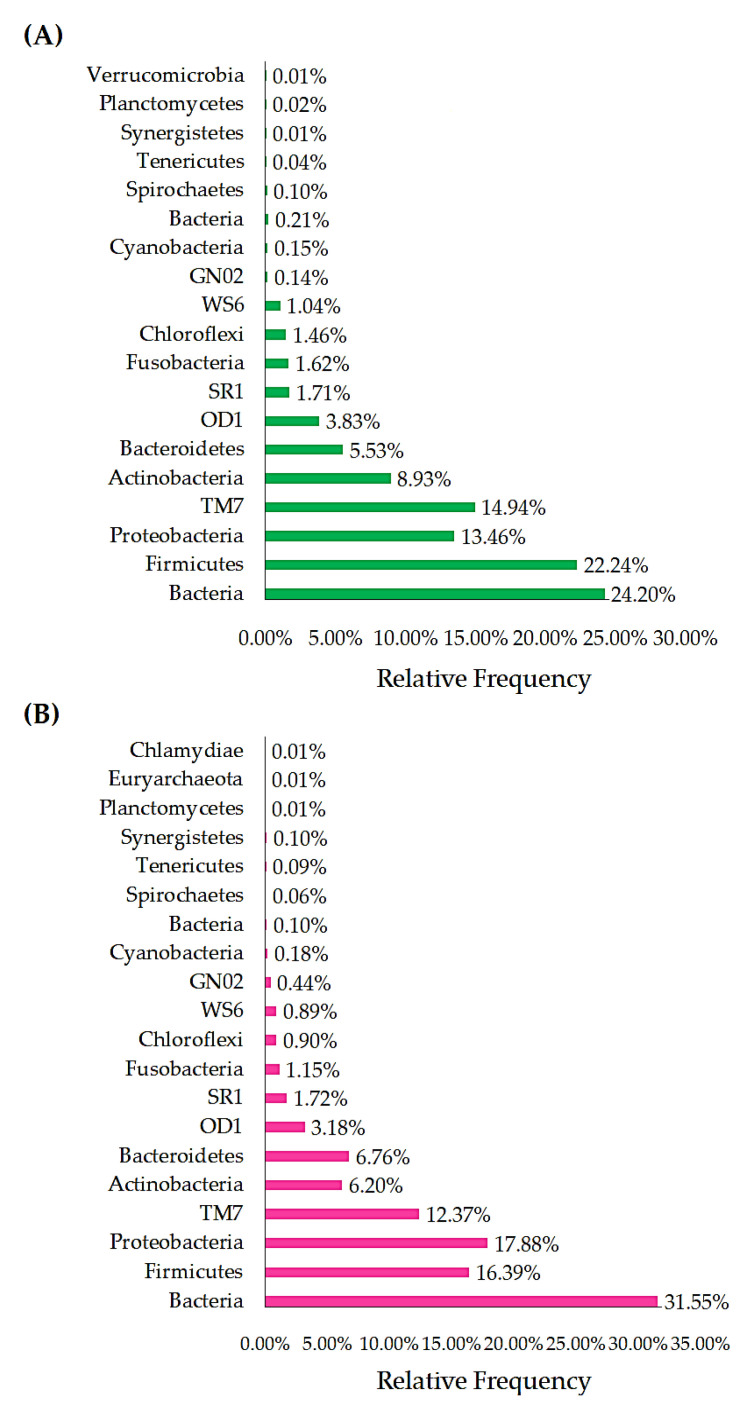
Comparison of the phylum-level relative frequency of PrTS (**A**) and PoTS (**B**) of the mouth-spray-treated group (G2). PrTS: pretreatment samples; PoTS: post-treatment samples.

Firmicutes (μ = 22.24%) was found majorly in the PrTS (Figure 3A), whereas the amount of Firmicutes (μ = 16.39%) was decreased in the PoTS (Figure 3B) of G2. However, the phylum Proteobacteria load (μ = 17.88%) was increased in the PoTS of G2 and it was identified as the major phylum. The phyla Bacteria (μ = 24.41%), Firmicutes (μ = 22.24%), Proteobacteria (μ = 13.46%), TM7 (μ = 14.94%), Actinobacteria (μ = 8.93%), Bacteroidetes (μ = 5.53%), OD1 (μ = 3.83%), SR1 (μ = 1.71%), Fusobacteria (μ = 1.62%), Chloroflexi (μ = 1.46%), WS6 (μ = 1.04%), GN02 (μ = 0.14%), Cyanobacteria (μ = 0.15%), Spirochaetes (μ = 0.10%), Tenericutes (μ = 0.04%), Synergistetes (μ = 0.01%), Planctomycetes (μ = 0.02%), and Verrucomicrobia (μ = 0.01%) were detected in the PrTS of G2 (Figure 3A). Meanwhile, the phyla Firmicutes (μ = 16.39%), TM7 (μ = 12.37%), Actinobacteria (μ = 6.20%), OD1 (μ = 3.18%), Fusobacteria (μ = 1.15%), Chloroflexi (μ = 0.90%), WS6 (μ = 0.89%), Spirochaetes (μ = 0.06%), and Planctomycetes (μ = 0.01%) amounts were decreased in the PoTS of G2. Moreover, the phyla Bacteria (μ = 31.65%), Proteobacteria (μ = 17.88%), Bacteroidetes (μ = 6.76%), SR1 (μ = 1.72%), GN02 (μ = 0.44%), Cyanobacteria (μ = 0.18%), Tenericutes (μ = 0.09%), and Synergistetes (μ = 0.10%) amounts were increased in the PoTS of G2. Euryarchaeota (μ = 0.01%) and Chlamydiae (μ = 0.01%) were observed in the PoTS of G2. However, the Verrucomicrobia was uprooted from the PoTS of G2 (Figure 3B).

#### 3.2.3. Genus

**Figure 4:**
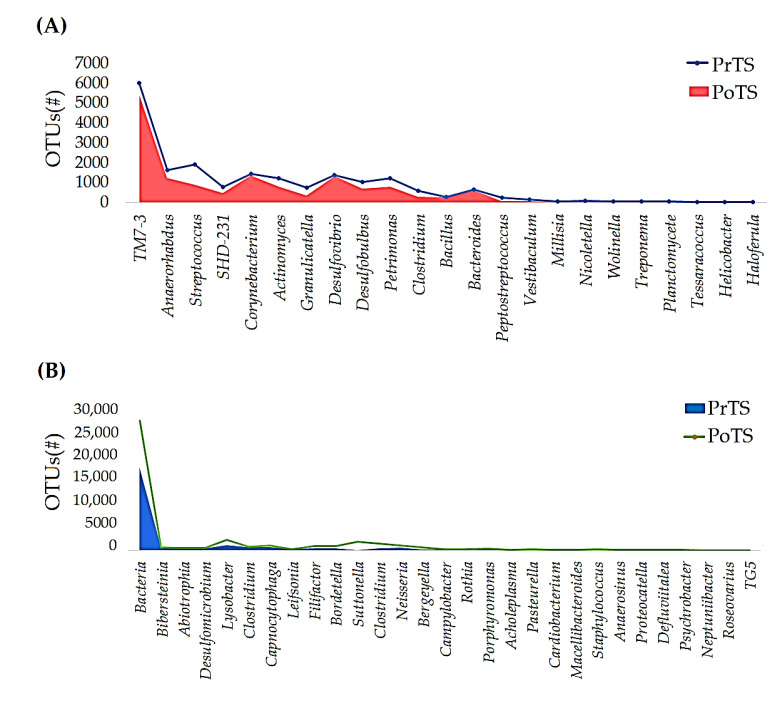

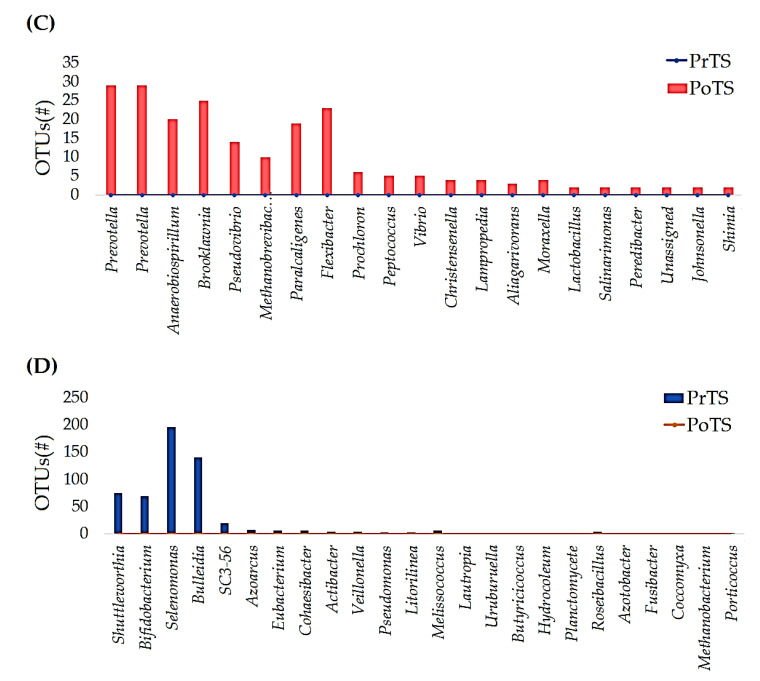
Comparison of the genus-level relative frequency of the PrTS and PoTS of G1. The reduced (**A**), increased (**B**), newly evolved (**C**), and completely vanished (**D**) genera in the control group after 42 days of study were reported. PrTS: pretreatment samples; PoTS: post-treatment samples.

The PrTS and PoTS represent 78 and 73 genera, respectively (Table S1). The richness of 23 genera, such as *TM7-3*, *Anaerorhabdus*, *Streptococcus*, *SHD*, *Corynebacterium*, *Actinomyces*, *Granulicatella*, *Desulfovibrio*, *Desulfobulbus*, *Petrimonas*, *Clostridium*, *Bacillus*, *Bacteroides*, *Peptostreptococcus, Vestibaculum*, *Millisia*, *Nicoletella*, *Wolinella*, *Treponema*, *Planctomycete*, *Tessaracoccus*, *Helicobacter*, and *Haloferula*, was reduced in the PoTS compared to the PrTS of G1 (Figure 4A). The richness of 29 genera was increased in PoTS of G1. Particularly, *Lysobacter*, *Suttonella*, *Clostridium*, *Capnocytophaga*, *Neisseria*, *Bordetella*, *Filifactor*, *Clostridium*, *Bergeyella*, and *Bibersteinia* levels were majorly increased (Figure 4B). Twenty genera, such as *Prevotella*, *Anaerobiospirillum*, *Brooklawnia*, *Pseudovibrio*, *Methanobrevibacter*, *Paralcaligenes*, *Flexibacter*, *Prochloron*, *Peptococcus*, *Vibrio*, *Christensenella*, ASW, *Lampropedia*, *Aliagarivorans*, *Moraxella*, *Lactobacillus*, *Salinarimonas*, *Peredibacter*, *Unassigned*, *Johnsonella*, and *Shimia*, were detected only in PoTS of G1 (Figure 4C). Noticeably, 24 genera vanished in the PoTS of G1 (Figure 4D).

**Figure 5:**
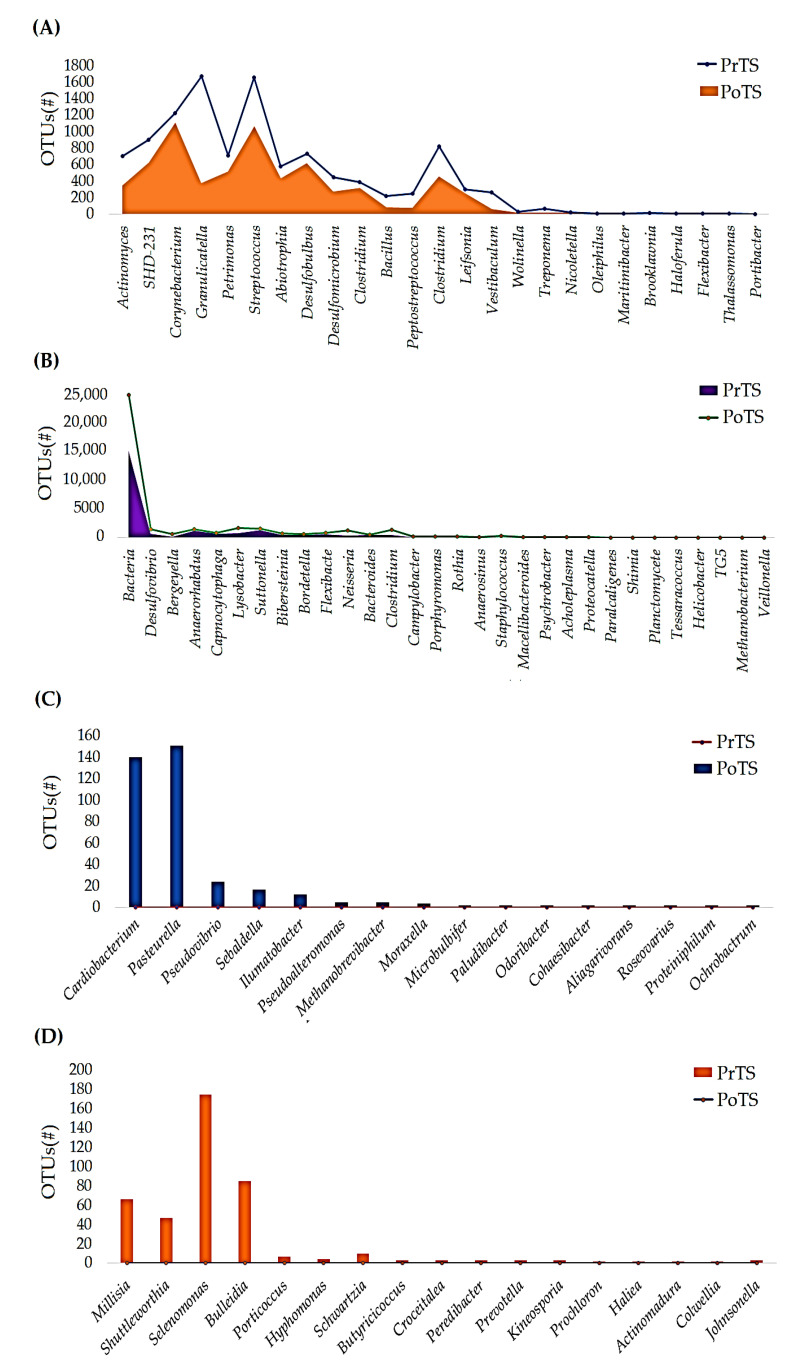
Comparison of the genus-level relative frequency of the PrTS and PoTS of G2. The reduced (**A**), increased (**B**), newly evolved (**C**), and completely vanished (**D**) genera in the control group after 42 days of study were reported. PrTS: pretreatment samples; PoTS: post-treatment samples.

About 73 and 71 genera were detected in the PrTS and PoTS of G2, respectively (Table S2). The abundances of 25e genera (*Actinomyces*, *SHD-231*, *Corynebacterium*, *Granulicatella*, *Petrimonas, Streptococcus*, *Abiotrophia*, *Desulfobulbus*, *Desulfomicrobium*, *Clostridium, Bacillus*, *Peptostreptococcus*, *Clostridium*, *Leifsonia*, *Vestibaculum*, *Wolinella*, *Treponema*, *Nicoletella, Oleiphilus*, *Maritimibacter*, *Brooklawnia*, *Haloferula*, *Flexibacter*, *Thalassomonas*, and *Portibacter*) were decreased (Figure 5A). In contrast, the richness of 30 genera was increased in the PoTS of G2 (Figure 5B). Sixteen genera (*Cardiobacterium*, *Pasteurella*, *Pseudovibrio*, *Sebaldella*, *Ilumatobacter*, *Pseudoalteromonas*, *Methanobrevibacter*, *Moraxella*, *Microbulbifer*, *Paludibacter*, *Odoribacter, Cohaesibacter*, *Aliagarivorans*, *Roseovarius*, *Proteiniphilum*, and *Ochrobactrum*) were identified only in PoTS (Figure 5C). At the same time, *Millisia*, *Johnsonella, Shuttleworthia, Selenomonas*, *Bulleidia*, *Porticoccus*, *Hyphomonas*, *Schwartzia*, *Butyricicoccus*, *Croceitalea*, *Peredibacter*, *Prevotella*, *Kineosporia*, *Prochloron*, *Haliea*, *Actinomadura*, and *Colwellia* vanished in PoTS of G2 (Figure 5D).

#### 3.2.4. Species

**Figure 6:**
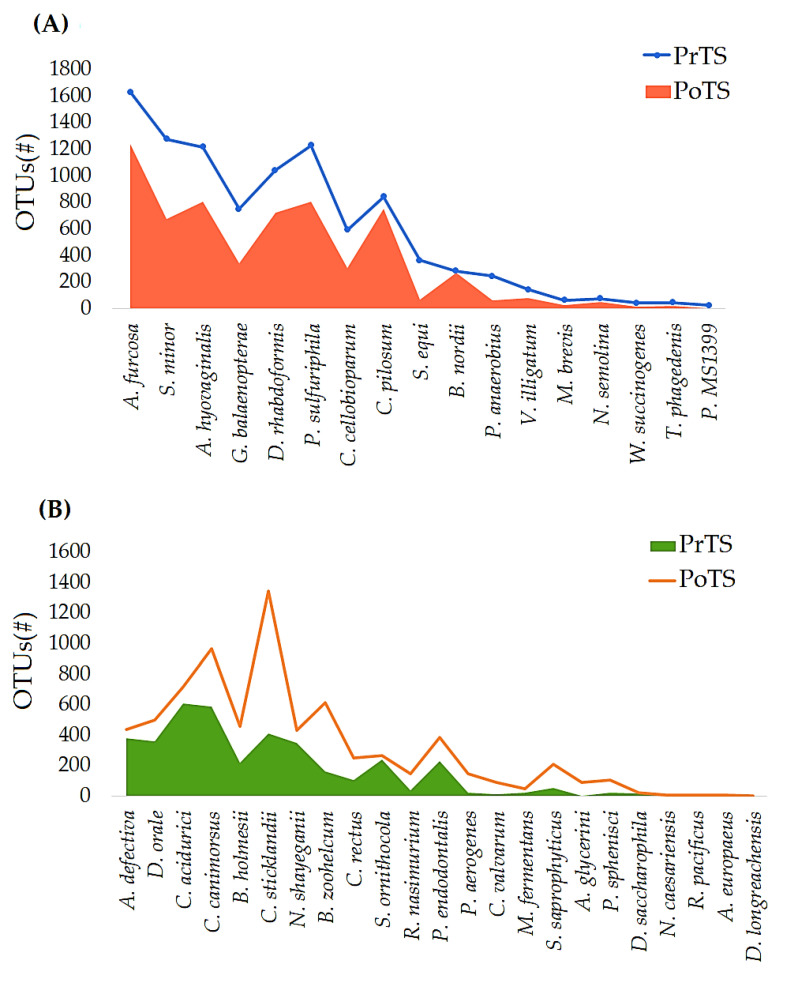

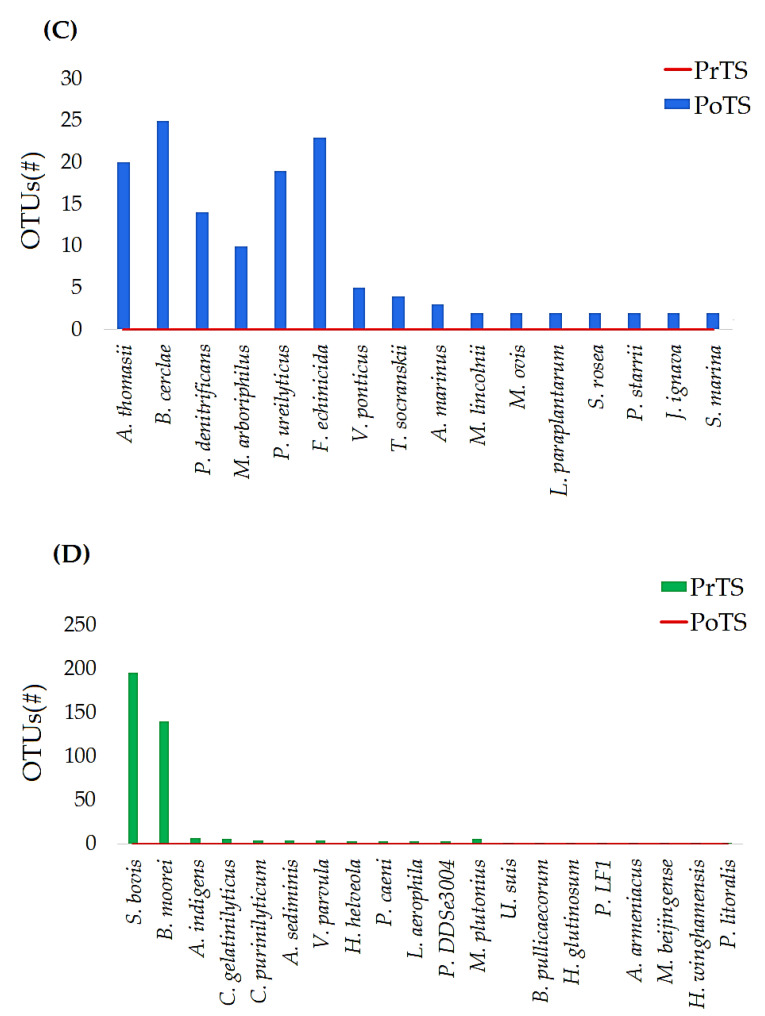
Comparison of the species-level relative frequency of the PrTS and PoTS of G1. The reduced (**A**), increased (**B**), newly evolved (**C**), and completely vanished (**D**) species in the control group after 42 days of study were reported.

About 62 and 57 species were detected in PrTS and PoTS of G1 (Table S3). The OTUs of 17 and 23 species were decreased (Figure 6A) and increased (Figure 6B), respectively, in PoTS of G1. *Anaerobiospirillum thomasii*, *Brooklawnia cerclae*, *Pseudovibrio denitrificans*, *Methanobrevibacter arboriphilus*, *Paralcaligenes ureilyticus*, *Flexibacter echinicida*, *Vibrio ponticus*, *Treponema socranskii*, *Aliagarivorans marinus*, *Moraxella lincolnii*, *Moraxella ovis*, *Lactobacillus paraplantarum*, *Salinarimonas rosea*, *Peredibacter starrii*, *Johnsonella ignava* (2), and *Shimia marina* were newly evolved in the PoTS of G1(Figure 6C). Meanwhile, *Selenomonas bovis*, *Bulleidia moorei*, *Azoarcus indigens*, *Cohaesibacter gelatinilyticus*, *Clostridium purinilyticum*, *Actibacter sediminis*, *Veillonella parvula*, *Haloferula helveola*, *Pseudomonas caeni*, *Litorilinea aerophile*, *Planctomycete DDSe3004*, *Melissococcus plutonius*, *Uruburuella suis, Butyricicoccus pullicaecorum*, *Hydrocoleum glutinosum*, *Planctomycete LF1*, *Azotobacter armeniacus*, *Methanobacterium beijingense*, *Helicobacter winghamensis*, and *Porticoccus litoralis* were completely rooted out in the PoTS of G1 (Figure 6D).

**Figure 7:**
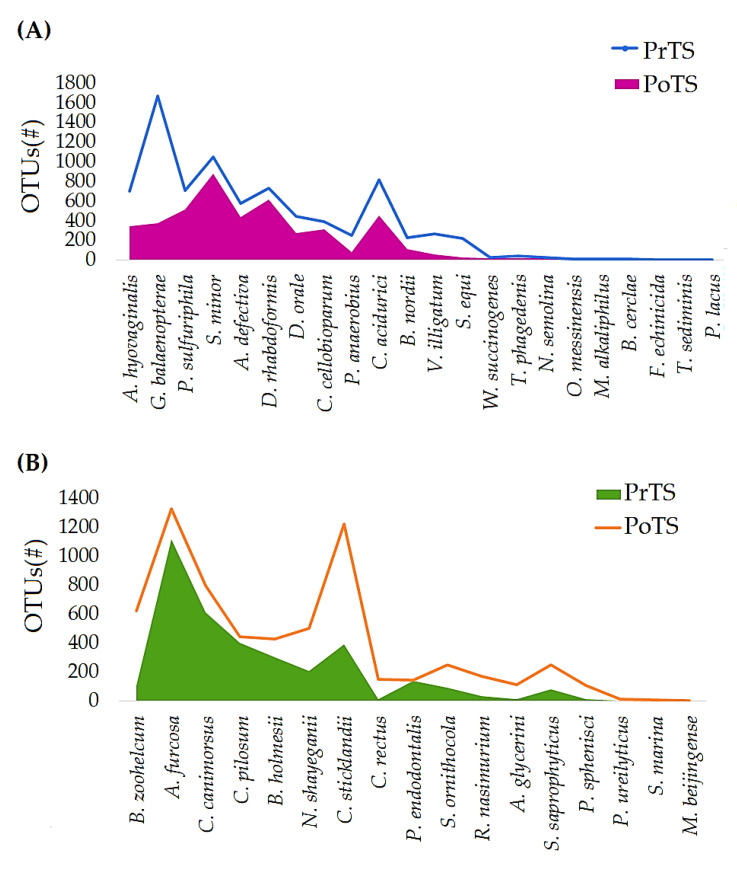

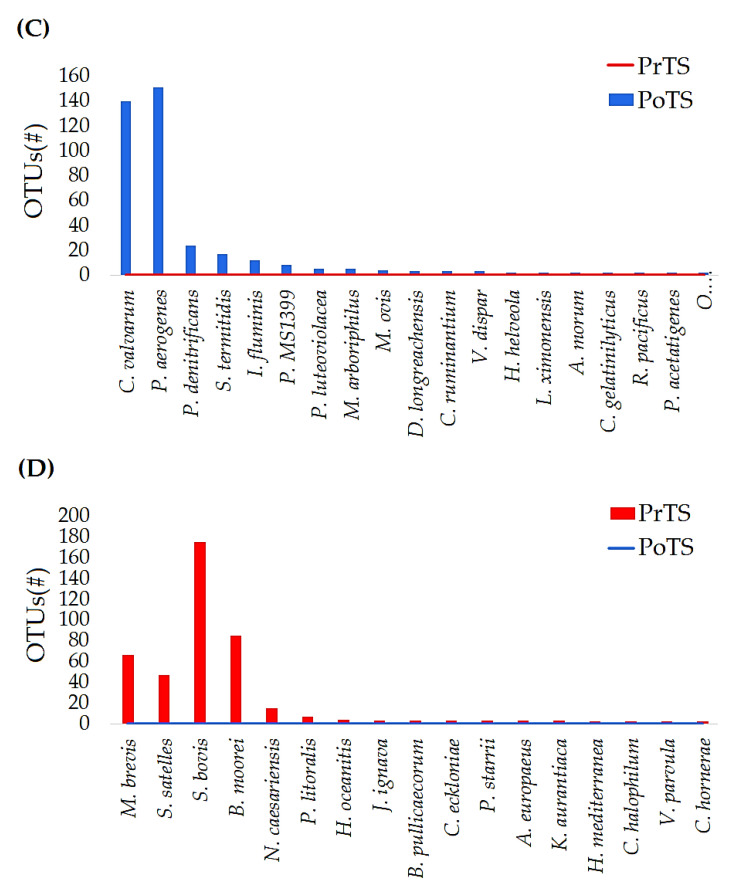
Comparison of the species-level relative frequency of the PrTS and PoTS samples of G2. The reduced (**A**), increased (**B**), newly evolved (**C**), and completely vanished (**D**) species in the control group after 42 days of study were reported.

About 57 and 59 species were detected in PrTS and PoTS of G2 (Table S4). The abundances of 22 and 17 species were decreased (Figure 7A) and increased (Figure 7B), respectively, in PoTS samples of G2. *Cardiobacterium valvarum*, *Pasteurella aerogenes*, *Pseudovibrio denitrificans*, *Sebaldella termitidis*, *Ilumatobacter fluminis*, *Planctomycete MS1399*, *Pseudoalteromonas luteoviolacea*, *Methanobrevibacter arboriphilus*, *Moraxella ovis*, *Desulfovibrio longreachensis*, *Clostridium ruminantium*, *Veillonella dispar*, *Haloferula helveola*, *Lysobacter ximonensis*, *Acholeplasma morum*, *Cohaesibacter gelatinilyticus*, *Roseovarius pacificus*, *Proteiniphilum acetatigenes*, and *Ochrobactrum pseudintermedium* were detected only in the PoTS of G2 (Figure 7C). Seventeen species completely vanished after mouth spray treatment (Figure 7D). The number of estimated phyla, genera, and species in the PrTS- and PoTS from the G1 and G2 groups were tabulated in Table S5.

### 3.3. Refraction Curve Analysis

#### 3.3.1. OTUs

**Figure 8:**
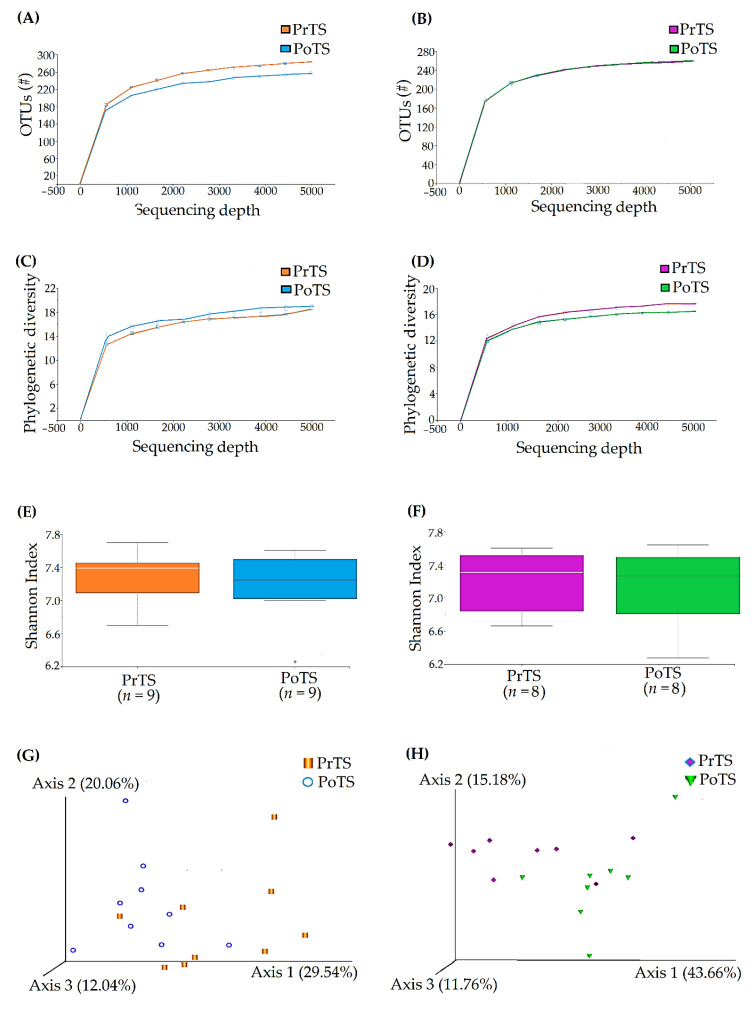
Refraction analysis for the G1 (PrTS and PoTS) and G2 (PrTS and PoTS) samples. The rarefaction curves of observed OTUs and the phylogenetic diversity were computed for G1 (**A**,**C**) and G2 (**B**,**D**). Shannon H-index alpha diversity of the G1 (**E**) and G2 (**F**) samples. The inter- and intragroup species differences of G1 (**G**) and G2 (**H**) samples were compared using PCoA.

In total, 429,943 and 430,482 16S rDNA V3-V4 sequencing reads were detected in the PrTS and PoTS of G1, respectively. After the quality control processing (filtration, denoising, and merging), about 67,848 and 81,420 (86.84%) high-quality nonchimeric reads were achieved for the PrTS and PoTS of G1, respectively. The reads were grouped into OTUs. About 215 and 210 OTUs were observed for each PrTS and PoTS, respectively (Figure 8A).

Likewise, 365,779 and 426,252 16S rDNA V3-V4 sequencing reads were obtained from the PrTS and PoTS of G2, respectively. After the quality control processing, around 61,335 and 72,914 high-quality nonchimeric reads were acquired for the PrTS and PoTS of G2, respectively. Then, the reads were clustered into OTUs. Averagely, each PrTS and PoTS of G2 had 247 and 258 OTUs, respectively (Figure 8B).

#### 3.3.2. Phylogenetic Diversity

The phylogenetic diversity was computed for the PrTS and PoTS of G1 and G2. The maximum phylogenetic diversity index for PrTS and PoTS of G1 were 18 and 19.5, respectively (Figure 8C). Similarly, the maximum phylogenetic diversity index for PrTS and PoTS of G2 were 16.5 and 15.6, respectively (Figure 8D).

#### 3.3.3. Species Richness

The species richness of the PrTS (Q1 = 6.90 and Q3 = 7.51) and PoTS (Q1 = 7.01 and Q3 = 7.49) treated samples of G1 was represented as quartile portions with the median of 7.27 and 7.19, and the whisker maximum of 7.69 and 7.60, respectively (Figure 8E). The species richness of the PrTS (Q1 = 6.77 and Q3 = 7.56) and PoTS (Q1 = 6.76 and Q3 = 7.58) treated samples of G2 was represented as quartile portions with the median of 7.31 and 7.27, and the whisker maximum of 7.60 and 7.64, respectively (Figure 8F).

#### 3.3.4. Principal Co-ordinates Analysis (PCoA)

The intragroup species differences between G1 and G2 samples were compared using PCoA. The phylogenetic diversity of G1 samples was represented as a PCoA plot, and the axis 1, 2, and 3 showed 29.54%, 20.6%, and 12.04% variations, respectively, which indicate the significant variations in the PoTS compared to PrTS of G1 (Figure 8G). Similarly, the axis 1, 2, and 3 showed 43.66%, 15.18%, and 11.76% variation, respectively, for G2 samples. The higher accumulation on axis 1 indicates the greater diversity in PoTS of G2 (Figure 8H).

## 4. Discussion

The GI was significantly (five out of eight teeth) reduced greatly in the G2. Similarly, PI was reduced in six out of eight teeth in G2 compared to the baseline values. The changes were not significant in the control (G1). Moreover, oral health was worsened in G1, regarding PI. The results indicate that the mouth sprays effectively improved oral health in GI and PI values (Table 2).

About 13,572 and 11,579 sequences were observed as differences in microbial sequences (PrTS vs. PoTS) in G1 and G2, respectively. The results indicate that mouth spray reduced the salivary microbial load in the cat (Figure 1A,B). According to the OTUs, phyla Firmicutes, TM7, Actinobacteria, Bacteroidetes, OD1, Fusobacteria, WS6, and Chloroflexi were reduced, and phyla Planctomycetes and Verrucomicrobia were completely eradicated in PoTS of the control (G1). The OTUs of the unclassified bacteria, Proteobacteria, SR1, GN02, and Spirochaetes, were greatly increased, and Tenericutes were newly evolved in the PoTS in G1 (Figure 2A,B). The phyla SR1 and GN02 are the common phyla in the cat and canine oral microbiome [17,27].

The total microbial sequences were increased after 42 days of usage of the mouth spray (72,914) compared to the baseline value (61,335). The bacterial lineage was increased in the PoTS of G2 (μ = 31.55%) compared to the PrTS of G2 (μ = 24.20%). The relative frequency of phyla Firmicutes, TM7, Actinobacteria, OD1, Fusobacteria, Chloroflexi, WS6, GN02, Spirochaetes, and Planctomycetes was reduced in G2, compared to baseline values (Figure 3A,B). The phylum Verrucomicrobia was completely eradicated, and phyla Euryarchaeota and Chlamydiae were newly detected in PoTS of G2 (Figure 3B).

*Chlamydia* is not associated with oral cat diseases [28,29]. Meanwhile, several Chlamydia species are transferable to humans and pose a substantial threat to public health, since they can end up causing pneumonia, atherosclerosis, coronary heart disease, and other serious illnesses [30,31].

The mouth spray containing gallic acid can reduce methane and nitrogen emissions in ruminants and suppress the growth of pathogens [32]. It is known that Proteobacteria and Actinobacteria are well-grown in the presence of nitrogen [33,34]. Fusobacteria is common in the oral cavity and connected to the early and late colonizing bacteria in dental plaque. It is involved in oral and extra-oral infections [35]. Rodrigues et al. and Abusleme et al. reported that the phyla Firmicutes, Fusobacteria, Syngeristetes, Chloroflexi, TM7, and Spirochaetes were more abundantly observed in the feline chronic gingivostomatitis and periodontitis-affected domestic cats [21,36]. The mouth spray containing hesperidin methyl chalcone was previously reported to inhibit oxidative stress and reduce the Fusobacteria growth [37]. The reduction in Fusobacteria was noticed in the present study.

The abundances of 23 and 29 genera were reduced and increased, respectively, in PoTS of G1, while 20 and 24 genera evolved and vanished, respectively (Figure 4A–D). The newly evolved *Prevotella* genus has been associated with oral cavity infections and peritonitis and is the most isolated anaerobe from pulmonary diseases and related consequences [38]. Methanogens are reported as pathogens linked to brain and muscle abscesses. In humans, they have been linked to oral microbiome dysbiosis, periodontitis, and peri-implantitis [39]. *Flexibacter* is a well-known fish pathogen that causes gill disease and egg and larvae disease, which causes some fish to die [40,41]. The genus *Johnsonella,* an opportunistic pathogen, was associated with chronic obstructive pulmonary disease but not periodontitis [42]. *Moraxella* species are pathogens that cause pediatric upper respiratory infections, such as otitis media, sinusitis, and pharyngitis [43]. The detection of new pathogens in PoTS of G1 showed that the untreated cats are susceptible to oral infections.

The mouth spray reduced the abundance of 25 genera and increased the load of 30 genera. About 17 genera completely vanished after 42 days of mouth spray usage in cats (Figure 5A–D). *Capnocytophaga, Pasteurella,* and *Bergeyella* are the dominant genus in healthy felines [21,22]. *Capnocytophaga* and *Bergeyella* growth was increased (Figure 5B); possibly, the mouth spray supported maintenance of the healthy oral microbiome in the cats. *Pasteurella* was described as the dominant genus in healthy cats [22] and was detected in the PoTS of G2 (Figure 5C). Oba et al. reported that *Desulfomicrobium* was enriched in adult dogs’ subgingival plaque [44].

*Desulfomicrobium* richness was decreased in the PoTS of G2 (Figure 5A). Moreover, the levels of *Abiotrophia* (associated with bacteremia and endocarditis) [45] and *Leifsonia* species (involved in oral infections) [46] were reduced in the PoTS of G2 (Figure 5A). *Schwartzia* was found in the subgingival plaque of the adult female Beagle dogs, and the mouth spray treatment completely eradicated the genus from the cats’ saliva (Figure 5D). *Moraxella* and *Neisseria* were reported as part of the normal oral flora of dogs and cats [47]. The genera (*Flexibacter, Prevotella,* and *Johnsonella*) found in the G1 were reduced or completely disappeared in the PoTS of G2 (Figure 5B,D). The results suggested that mouth spray reduced the harmful microbes and supported the growth of beneficial bacteria in cats.

*Actinomyces hyovaginalis* is associated with human and bovine diseases, commensals of healthy Nubian goats [48]. *Desulfomicrobium orale* is involved in human periodontal disease [49]. *Nicoletella semolina* was abundantly detected in healthy horses and those with severe asthma. However, the pathogenicity is not yet elucidated [50]. *A. hyovaginalis*, *D. orale*, and *Nicoletella* species abundances were lowered in the PoTS of G2 compared to baseline values (Figure 7A), which showed that the mouth spray could reduce the opportunistic pathogens’ load in cats.

The OTUs of the PrTS of G2 were lower than the PoTS of G1. After 42 days, the OTUs were reduced further in the G2 samples. The results revealed that the microbial diversity was decreased in the mouth-spray-treated samples (Figure 8A,B). The phylogenetic diversity showed that the mean microbial diversity was higher in PoTS of G1, while, in G2, diversity was less than baseline values (Figure 8C,D). The species richness was reduced in PoTS of both G1 and G2 (Figure 8E,F). PCoA analysis suggested that mouth spray significantly affected the microbial diversity of healthy cats (Figure 8G,H). The microbiota results were consistent with the outcomes of clinical parameters (GI and PI).

## 5. Conclusions

The current study describes the changes in the salivary microbial diversity and the impact of gallic acid-containing mouth spray on the oral health of healthy cats. The commensal microbial load (*Porphyromonas* and *Moraxella*) was increased in mouth-spray-treated cats. The mouth spray efficiently acts against pathogens, especially *D. orale*. The microbiota changes were consistent with the outcomes of clinical parameters, such as GI and PI. The studied mouth spray could maintain a healthy oral microbiome, and it could be used as an adjuvant medication to treat the cats’ oral diseases. However, further studies are needed to confirm the therapeutic potential of the mouth spray.

## Figures and Tables

**Table 1:** Demographic data of the experimental cats.

Demographic	Control	Treatment	*p*-Value
Male (n; %)	11 (55)	7 (35)	0.341 *
Female (n; %)	9 (45)	13 (65)	
Weight (Kg; Mean ± SD)	4.46 ± 1.07	4.23 ± 1.00	0.285 **
Age (Year; Mean ± SD)	5.00 ± 1.67	4.64 ± 1.68	0.622 **

* Fisher’s exact test; ** Mann–Whitney U test.

**Table 2:** Median, interquartile values, and comparison (pre- vs. post-treatment) of the gingival index (GI) and plaque index (PI) for control and treatment groups.

Para-Meters	Tooth Code	Median (Interquartile Range: Q3–Q1)					
		Control			Treatment		
		Pre (Day 0)	Post (Day 42)	Comparison * *p*-Value	Pre (Day 0)	Post (Day 42)	Comparison * *p*-Value
GI	104	1 (2)	0.5 (1)	0.1255	0 (1)	0 (1)	0.2334
	108	2 (0.5)	1 (2)	0.0053 (−)	2 (1)	0.5 (1)	0.0003 (−)
	204	0.5 (2)	1 (1)	0.8162	0 (2)	0 (1)	0.3223
	208	2 (0)	1 (1)	0.0539	2 (1)	1 (2)	0.0111 (−)
	304	1 (2)	0 (1)	0.0843	0 (1)	0 (0.5)	0.0327 (−)
	309	1 (2)	1 (2)	1.00	1 (2)	0 (1.5)	0.0122 (−)
	404	1 (1.5)	0 (1.5)	0.4401	0 (1)	0 (0)	0.4705
	409	1 (1)	1 (1.5)	0.0766	1 (2)	0 (1)	0.0196 (−)
PI	104	1 (1.5)	1.5 (1)	0.0231 (+)	1 (2)	1 (1)	0.0497 (−)
	108	2 (1)	2 (2)	0.7243	3 (1)	1.5 (1.5)	0.0154 (−)
	204	1 (2)	2 (2)	0.0237 (+)	2 (1.5)	1 (1)	0.0084 (−)
	208	2 (2)	3 (1)	0.1968	3 (1)	3 (1)	0.9319
	304	1 (1)	1 (1)	0.0155 (+)	1 (0.5)	1 (0)	0.0257 (−)
	309	1 (2)	2 (1)	0.0699 (+)	2 (1.5)	1 (1)	0.0298 (−)
	404	1 (1)	1 (0.5)	0.0004 (+)	1 (0)	1 (0)	0.3173
	409	1 (2)	1 (1)	0.251 (+)	2 (2)	1 (1)	0.0375 (−)

* Wilcoxon signed-rank test. Significance level (α): *p* ≤ 0.05. − and + signs indicate the significant decrease and increase in the factors, respectively.

## Data Availability

All the related data have been provided in the manuscript.

## Supplementary Materials

The following supporting information can be downloaded at: https://www.mdpi.com/article/10.3390/vetsci9070313/s1, Table S1: The number of genera identified from the Pre- and Post-treated samples of G1; Table S2: The number of genera identified from the pre-and post-treated samples of G2; Table S3: The number of species identified from the pre-and post-treated samples of G1; Table S4: The number of species identified from the pre-and post-treated samples of G2; Table S5: The number of phylum, genus and species estimated in the pre and post-treated samples of G1 and G2.
